# Global detection of human variants and isoforms by deep proteome sequencing

**DOI:** 10.1038/s41587-023-01714-x

**Published:** 2023-03-23

**Authors:** Pavel Sinitcyn, Alicia L. Richards, Robert J. Weatheritt, Dain R. Brademan, Harald Marx, Evgenia Shishkova, Jesse G. Meyer, Alexander S. Hebert, Michael S. Westphall, Benjamin J. Blencowe, Jürgen Cox, Joshua J. Coon

**Affiliations:** 1 Computational Systems Biochemistry Research Group, Max Planck Institute of Biochemistry, Martinsried, Germany; 2 Morgridge Institute for Research, Madison, WI USA; 3 National Center for Quantitative Biology of Complex Systems, University of Wisconsin-Madison, Madison, WI USA; 4 Department of Chemistry, University of Wisconsin-Madison, Madison, WI USA; 5 EMBL Australia and Garvan Institute of Medical Research, Sydney, New South Wales Australia; 6 School of Biotechnology and Biomolecular Sciences, University of New South Wales, Sydney, New South Wales Australia; 7 Department of Biomolecular Chemistry, University of Wisconsin-Madison, Madison, WI USA; 8 Department of Microbiology and Ecosystem Science, University of Vienna, Vienna, Austria; 9 The Donnelly Centre, University of Toronto, Toronto, Ontario Canada; 10 Department of Molecular Genetics, University of Toronto, Toronto, Ontario Canada

**Keywords:** Proteomics, Proteome informatics, Data integration

## Abstract

An average shotgun proteomics experiment detects approximately 10,000 human proteins from a single sample. However, individual proteins are typically identified by peptide sequences representing a small fraction of their total amino acids. Hence, an average shotgun experiment fails to distinguish different protein variants and isoforms. Deeper proteome sequencing is therefore required for the global discovery of protein isoforms. Using six different human cell lines, six proteases, deep fractionation and three tandem mass spectrometry fragmentation methods, we identify a million unique peptides from 17,717 protein groups, with a median sequence coverage of approximately 80%. Direct comparison with RNA expression data provides evidence for the translation of most nonsynonymous variants. We have also hypothesized that undetected variants likely arise from mutation-induced protein instability. We further observe comparable detection rates for exon–exon junction peptides representing constitutive and alternative splicing events. Our dataset represents a resource for proteoform discovery and provides direct evidence that most frame-preserving alternatively spliced isoforms are translated.

## Abstract (web summary)

Deep proteome sequencing achieves ~80% coverage of the human proteome.

## Main

Near-complete proteomes of simple organisms can be detected by mass spectrometry (MS) following only 1 h of analysis^1,2^. For more complex organisms, it is possible to monitor over 10,000 proteins within a day (refs. ^3–7^). Community-based maps of the human proteome, assembled using extensive data from various tissues and cell types from laboratories across the world, have provided evidence for the translation of >90% of annotated protein-coding genes^7,8^. However, although the human genome contains approximately 20,000 protein-coding genes^9,10^, it is estimated that alternative splicing events, whereby precursor messenger RNA sequences are combined in different arrangements, have the potential to notably increase proteome diversity. Specifically, from RNA sequencing (RNA-seq) analysis of human organs, reports have estimated that transcripts from more than 95% of multi-exon genes undergo alternative splicing^11,12^. Furthermore, recent single-cell transcriptome sequencing has revealed that true splice isoform complexity is likely greater than previously appreciated^13,14^. Other sources of proteome variation, such as single-amino acid polymorphisms (SAPs), alternative splicing and posttranslational modifications, further increase proteomic complexity^15–20^.

Limitations in proteomic technology have not permitted the global-scale detection of protein diversity. Typically, for shotgun proteomic methods, the presence of an entire protein is determined using a small number of peptide proxies—as few as two or three. Thus, sequence coverage in a proteomics experiment is generally insufficient to fully characterize all protein states present within a sample^21,22^. Yet the ability to precisely monitor protein isoforms is essential to understanding biological systems. Even the current deepest proteomic datasets^23,24^ do not contain enough sequence data to globally identify proteoforms. One approach to achieving proteoform-level detection is top-down MS, a strategy that measures intact protein mass before dissociation for sequence determination using tandem mass spectrometry (MS/MS). Ensuring no loss in resolution, the top-down strategy is appealing. Practical issues with high-mass proteins, sequence coverage and detection of low-abundance species, however, limit its impact^25^.

Given the technical hurdles with top-down proteomics, we revisited the shotgun strategy. Shotgun proteomics preferentially relies on trypsin to catalyze hydrolysis of proteins. Trypsin cleaves C-terminal to lysine and arginine residues and produces peptides of length and charge distributions most amenable to MS/MS. However, even with the assistance of extensive chromatographic separation, not all portions of the proteome are accessible from tryptic peptides^26,27^; many of the peptides produced are either too short or too long to be detected using current liquid chromatography–mass spectrometry (LC–MS) technology. As proteoforms can differ by a small number of amino acids, extensive sequence coverage is crucial for distinguishing near-identical variants. The use of alternative enzymes in addition to trypsin during digestion can increase the amino acid coverage of individual proteins, phosphorylation sites and whole proteomes^28–44^. However, given the considerably increased effort involved, this strategy is not amenable to routine use and to our knowledge has not been previously employed for the global-scale detection of proteoforms.

In this study, we investigate whether the separate digestion of human proteomes expressed in six different cell lines with six different proteases, coupled with extensive liquid chromatography (LC) fractionation and state-of-the-art MS, produces sufficient sequence depth to afford a global assessment of how genomic variants and alternative splicing are incorporated into the proteome. Generated peptides were extensively fractionated before analysis on an Orbitrap Tribrid mass spectrometer, where they were dissociated using various fragmentation methods, including higher-energy collisional dissociation (HCD)^45^, collisionally activated dissociation (CAD)^46^ and electron transfer dissociation (ETD)^47,48^. We collected ~20 million high-resolution mass spectra and ~164 million MS/MS spectra from ~2,500 nano-scale liquid chromatography-tandem mass spectrometry (nLC–MS/MS) experiments. The combined data enabled identification of 17,717 unique proteins with an overall median sequence coverage of 79.2%. Using these data, we provide a global view of genomic and transcriptomic sequence variant expression at the protein level. From a direct comparison with quantitative RNA-seq data, we detect ~80% of SAPs and ~20% of exon–exon junctions, representing both inclusion and skipping of frame-preserving alternative splicing events. However, for proteins with the highest proteomics sequence coverage, represented by genes with relatively high expression (that is, log_2_ of reads per kilobase per million (RPKM) of ≥7) at the transcript level, ~64% of frame-preserving alternatively splicing events are detected and the rates of detection of constitutively spliced and alternatively spliced junctions are similar. And finally, using the extensive, overlapping peptide sequence information provided by this resource, we demonstrate the feasibility of de novo protein assembly. Data generated from the present study represent the deepest proteomics map collected to date and have been compiled into an online resource at deep-sequencing.app. These methods and resources lay the foundation for comprehensive mapping of protein diversity and are expected to catalyze future research efforts.

## Results

### Deep human proteome sequencing

In silico tryptic digestion of the ~21,030 reviewed canonical protein sequences of the human proteome (UniProtKB/Swiss-Prot) predicts 2.3 million tryptic peptides of suitable size for MS detection (7–35 amino acids, up to two missed cleavages). These peptides comprise 9.9 million amino acid residues of the 11.5 million total—that is, only 86% of the proteome. If we consider digestion of the same proteins using the six enzymes in our study (LysC, LysN, AspN, chymotrypsin, GluC and trypsin), 7.4 million peptides suitable for shotgun proteomics are generated. These peptides cover 99% of the amino acids contained in the human proteome.

**Fig. 1:**
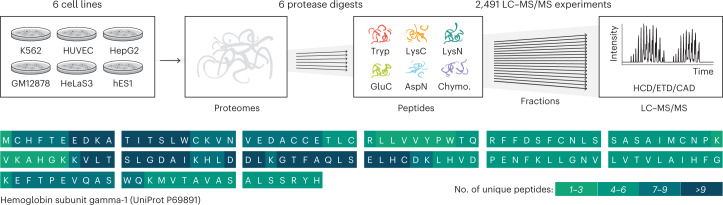
Deep proteome sequencing workflow. Six human cell lines were grown in parallel, their proteomes were isolated and then one of the six proteases was used to digest separate aliquots of each proteome in parallel. Peptides resulting from each digestion were fractionated by high-pH RP chromatography and then analyzed separately with nLC–MS/MS using HCD, ETD and CAD. The resulting data were searched with MaxQuant^51,52^ against the human proteome database, and over 17,000 proteins were identified by peptides that produce a median coverage of over 80%. The high coverage achieved is illustrated on the sequence of hemoglobin subunit gamma-1, with color coding to illustrate the number of unique peptides that cover each amino acid position.

To test the hypothesis that we can in such manner increase coverage of the human proteome, we selected six diverse human cell lines: hES1, an embryonic stem cell line; HeLa S3, from cervical carcinoma; HepG2, from liver carcinoma; GM12878, a blood lymphoblastoid line; K562, from chronic myeloid leukemia; and HUVEC, from umbilical vein epithelial cells (Fig. 1). Having been included in the Encyclopedia of DNA Elements (ENCODE) project, these cell lines have a large amount of publicly available genomic and transcriptomic data^49^. Proteins from each cell line were separately digested with the six proteases listed above. To maximize depth, the resultant peptides were heavily fractionated (24–80 fractions) and analyzed using nano flow LC coupled with quadrupole-Orbitrap–linear ion trap hybrid MS systems. Dissociation for MS/MS was achieved using HCD, CAD and ETD. The resulting 2,491 raw files were simultaneously analyzed by database search to identify proteins and peptides using the Andromeda search engine^50^ inside MaxQuant^51,52^, and results were sequentially filtered to 1% peptide spectrum matches (PSMs) and protein-level false discovery rate (FDR) over the whole dataset.

**Fig. 2:**
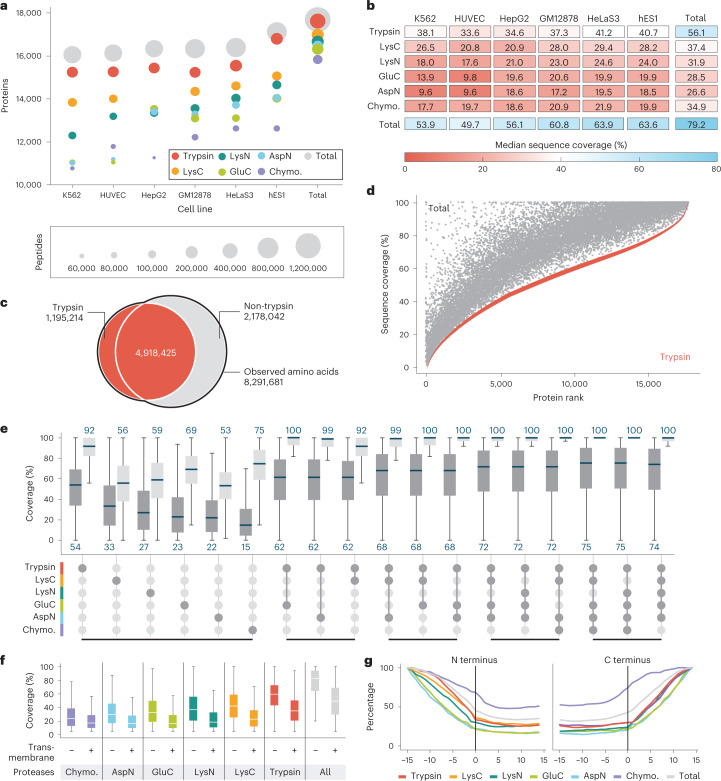
Overview of results from deep proteomics analysis. **a**, Number of proteins detected for each of the six cell lines and cumulative as a function of peptides from the various protease digests. **b**, Median sequence coverage of various cell line proteomes achieved by digests with individual proteases and by combining all protease results. Supplementary Fig. 2c shows sequence coverage distributions separately for all combinations of cell lines, proteases and fragmentation methods. **c**, Venn diagram of all observed amino acids digested by trypsin versus all proteases combined excluding trypsin. **d**, Sequence coverage for each of the detected proteins for the tryptic peptide data (red) and combined protease digests, including trypsin (gray). **e**, Observed (dark gray) and theoretical (light gray) distributions of sequence coverage achieved for various combinations of proteases. The top three combinations of 2, 3, 4 or 5 proteases are displayed. **f**, Protein coverage comparison of transmembrane and nonmembrane proteins. For **e** and **f**, the lower whisker/quartile and upper quartile/whisker show the 5th, 25th, 75th and 95th percentiles, accordingly. **g**, Relative protein coverage of N terminus (left) and C terminus (right) transmembrane segments. Chymo., chymotrypsin.

Figure 2 summarizes these data, showcasing the depth of coverage and gains achieved by the multi-enzyme approach. For each cell line, an average of 539,325 unique peptides, corresponding to ~16,000 proteins, were identified (Fig. 2a). The highest number of identified proteins was from the hES1 cell line (17,121), followed by HeLa S3 (16,399), GM12878 (16,344), HepG2 (16,328), HUVEC (16,158) and K562 (16,054). The trypsin dataset contributed the largest number of unique peptides (396,782), followed by LysN (194,506), LysC (193,956), GluC (162,784), AspN (152,259) and chymotrypsin (114,152). Properties of detected peptides, such as a number of missed cleavages, length distribution and cleavage motif, are in high agreement with previous proteomics multi-enzyme studies (Supplementary Fig. 1)^26,27,37^. Notably, within each cell line, data from each enzyme digestion alone identified over 10,000 protein groups. Data from tryptic peptides contributed the largest number of identifications and unique sequences, totaling 17,631 proteins with 56.5% median sequence coverage. However, using all data comprising all proteases afforded a modest increase in the number of identified proteins (17,717) but considerably boosted the median sequence coverage to 79.2%. In total, we identified 12,151,708 PSMs and 1,119,510 unique peptides at FDR of 1%. Of those, 790 proteins were identified with complete sequence coverage. The average number of unique peptides per protein was 97 (median 65). However, 54 proteins were identified by only one unique peptide; only 1,122 proteins, or 6.3% of the total proteins, were identified by ten or fewer unique peptides. Median sequence coverage for the combined dataset and the contribution from subsets is shown in Fig. 2b, and ranges from 49.7% (HUVEC; 16,158 proteins) to 63.9% (HeLa S3; 16,399 proteins). Remarkably, nearly half of all identified proteins were observed with 80–100% sequence coverage (Supplementary Fig. 2a,b). Only 936 proteins, or 5.3% of the total data, have sequence coverage below 25%.

The addition of enzymes other than trypsin provided a slight increase in the total number of proteins identified but induced a large increase in the nonredundant amino acids detected. The 17,717 detected human proteins comprise 12,006,700 amino acid residues, including those that arise from noncanonical proteins, that is, isoforms. In total, the unique peptides identified in the combined tryptic datasets from all cell lines detected approximately half of these amino acids (6,113,639). The number of covered amino acids rises to 8,291,681 when all protease data are used (Fig. 2c). Figure 2d illustrates the impact of these additional amino acids on protein sequence coverage. Next, we determined the most optimal multi-protease combinations (Fig. 2e), noting that all top combinations included trypsin. Our total human proteome coverage is, to our knowledge, the largest to date, with 2.12 million more residues (a 34.4% increase) over the 6.17 million identified using exclusively tryptic peptides from the entire MassIVE data repository (Supplementary Fig. 2d)^8^. Finally, we compared the proteins identified in this study with the curated neXtProt database^7^, which categorizes proteins across five groups based on the strength of the evidence for their existence. As shown in Supplementary Fig. 3, most of our protein identifications (13,603 proteins) fall into the highest-confidence category (PE1), and 79 proteins now can be promoted to PE1 status from lower categories (Supplementary Table 1).

Alternative proteases have previously been utilized to uncover novel portions of the proteome, including membrane proteins^53,54^. These proteins—essential to many biological processes and representing important drug discovery targets^55^—remain under-represented in proteomics datasets due to their hydrophobic nature. This is also true of our dataset. Gene ontology cellular component pathway enrichment analysis of the proteins with sequence coverage below 25% revealed that these low-coverage proteins were primarily membrane proteins (Supplementary Fig. 2e). Indeed, we also observe a coverage reduction for transmembrane proteins across all studied proteases (Fig. 2f). To further explore the behavior of peptides generated from transmembrane-spanning sequences, we calculated the enzyme-specific coverage of aligned membrane-spanning regions to either the N or C terminus (Fig. 2g). These data demonstrate that because transmembrane regions are depleted for typical protease cleavage sites, peptides suitable for detection by shotgun proteomics are less likely to be observed. This conclusion is further supported by the strong relative performance of chymotrypsin, which is atypical in cleaving at hydrophobic residues, as compared with the other proteases.

### De novo protein assembly

Protein inference is conceptually akin to reference transcriptome assembly in short-read sequencing, where a previously assembled proteome or genome database is required to map peptide sequences or nucleic acid reads, respectively. In proteomics, however, genome assemblies for proteome database generation are either unavailable or low-quality for many organisms. Several tools are available to assemble short sequencing reads without a reference genome, such as SOAPdenovo-Trans^56^. However, de novo assembly of nucleic acid sequences relies on the presence of randomly overlapping sequences, which is not a common property of proteomic datasets, which typically use only a single enzyme (for example, trypsin).

With the data from six different proteases and deep coverage presented above, we produce many peptides with partial overlap, which we hypothesized may enable de novo protein assembly. An excellent example for the de novo assembly is the proteasome subunit alpha type-6, which is represented by full sequence coverage (Supplementary Fig. 4a). Overall, the de novo assembly produced 35,480 scaffolds, of which 16,496 (~47%) correctly match to 9,695 protein groups. Median sequence coverage from the de novo assembly was 18% compared with 79.2% for the reference assembly (Supplementary Fig. 4b,c). Assembled scaffolds have a range of 33–358 amino acids with a median length of 45 (Supplementary Fig. 4d), and an average of two scaffolds were mapped to each protein (Supplementary Fig. 4e). These results demonstrate the feasibility of de novo proteome assembly using overlapping peptides from multiple protease digestions of the proteome; application of proteomics-specific assembly methods may improve this result in the future^57^.

### Majority of hypothetical SAPs are confirmed in the proteome

**Fig. 3:**
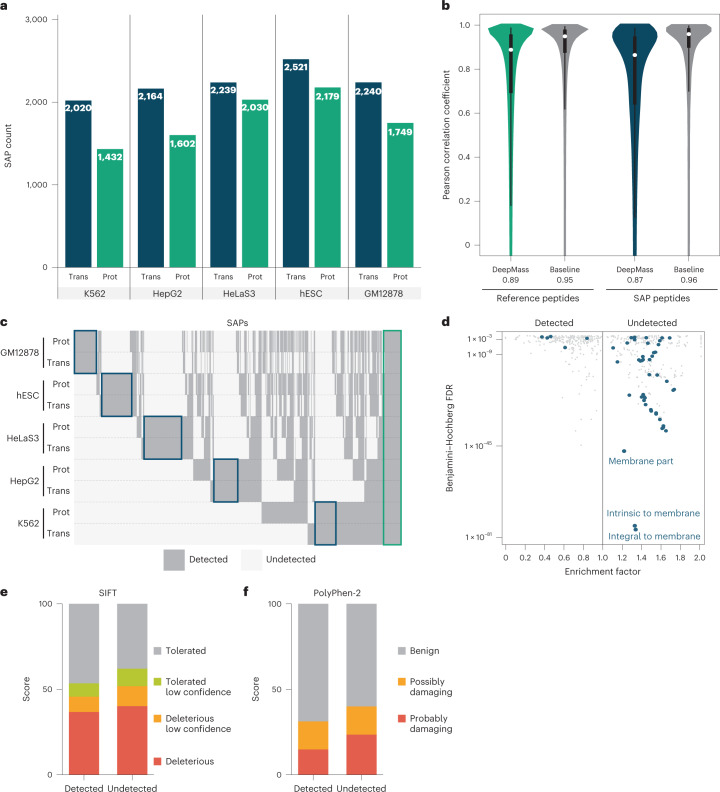
Discovery of proteins with SAPs. **a**, Comparison of SAPs discovered in the ENCODE transcriptomic data (Trans) and presented proteomics data (Prot) for each of the cell lines. **b**, Distribution of correlation coefficients between observed and predicted by DeepMass^60^ spectra. The baseline distribution shows acquisition-to-acquisition variation by comparing observed spectra for peptides. The white circle shows the median value. The lower and upper quartiles of the box demonstrate the 25th and 75th percentiles, accordingly. The lower and upper whiskers show the 5th and 95th percentiles, accordingly. The distributions are based on 5,128,969, 442,476, 16,516 and 4,969 comparisons (from left to right). **c**, Clustered binary heatmap of the detected SAPs row-grouped by cell line and omics platform (transcriptomics or proteomics). Blue rectangles highlight clusters specific to each cell line, and the green rectangle SAPs that are conserved across all cell lines. **d**, Gene ontology (GO) enrichment of genes with SAPs detected or undetected by MS. Genes with a mixed population of SAPs were removed, and repeats collapsed. Blue dots highlight GO terms with the word ‘membrane’ mentioned in the name. **e**. SIFT-generated^61^ score distribution over four categories for detected and undetected SAPs. Applying the two-sided Wilcoxon rank sum test on the raw scores results in *P* value of 2 × 10^−8^. **f**, The same as **e**, but for the PolyPhen-2 (ref. ^62^) tool. Applying the two-sided Wilcoxon rank sum test on the raw scores results in *P* value of 1.1 × 10^−12^.

SAPs are variations in the protein sequence which often arise from single nucleotide polymorphisms (SNPs) that result in nonsynonymous codon changes in genomic sequence. The HeLa S3 cell line used in this study contains ~4.5 million SNPs when compared with the hg38 reference human genome. Of these, ~30,000 occur in coding regions, and 4,740 result in nonsynonymous codon changes^58^. We assessed whether our deep proteomics data would afford the ability to determine whether these SNPs are translated into SAPs. To this end, we searched for SAPs with a MaxQuant module which is tailored for the identification of peptide evidence for the translation of genomic variations (Supplementary Fig. 5)^59^. From this analysis, we observe protein-level evidence for up to 2,179 SAPs in individual cell lines, or a total of 5,060 SAPs (Fig. 3a and Supplementary Table 2). To assess the quality of these SAP-containing peptide identifications, we performed a correlation analysis of all peptide spectral matches both with and without SAPs (mutated and reference peptides, respectively). Figure 3b demonstrates the distribution of correlation coefficients between observed and predicted MS/MS spectra using the machine learning-based tool DeepMass^60^ for mutated and reference peptides. The baseline is drawn for peptides with multiple fragmentation spectra, which are compared with each other. The distributions for reference and mutated peptides are similar, providing increased confidence that these peptide spectral matches are legitimate.

For all cell lines except HUVEC, we observed high overlap between the mutations detected by transcriptomics and by proteomics (Supplementary Fig. 6a). Given HUVEC is the only primary cell line (that is, obtained directly from host tissue) in the study, this low overlap is expected as the transcriptomic and proteomic data were collected from cells originating from different donors. Therefore, we omitted HUVEC from further analysis. Figure 3a shows that most nonsynonymous SNPs that appear in the transcript also appear at the protein level (median 73% over all studied cell lines). Further, the multi-enzyme data led on average to a doubling of identified SAPs compared with when only trypsin was used (Supplementary Fig. 6a).

Figure 3c shows the presence of variants as a function of cell line and whether they are detected at the protein level. We note that there are primarily two types of SAP—those that are cell line specific (highlighted within a blue rectangle) and those that are conserved across the cell lines (highlighted within a green rectangle). Enrichment analysis of the SAPs found only at the transcriptomic level (Fig. 3d) revealed several gene ontology terms associated with membrane protein families—supporting our earlier conclusions that peptides for such proteins are less amenable to MS analysis.

To test whether some of the mutations that were undetected at the protein level, even though transcripts evidence was present, caused protein instability, we leveraged the SIFT^61^ and PolyPhen-2 (ref. ^62^) tools. These software tools predict how an amino acid mutation can alter protein structure and function by classifying mutations as either benign or deleterious. As depicted in Fig. 3e,f, both algorithms predict a significant shift (*P* values of 2 × 10^−8^ and 1.1 × 10^−12^, respectively from two-sided Wilcoxon rank sum test) in the fraction of deleterious mutations for the undetected SAP group. These data confirm that at least a subset of undetected SAPs likely arise from cases where the mutation induces protein instability.

### Protein-level evidence for alternative splicing

The high proteome sequence coverage of our dataset provides an opportunity to globally detect protein isoforms arising from alternative splicing and affords a direct assessment of the degree to which this process contributes to proteomic complexity. As mentioned above, RNA-seq analyses of diverse human organs and cell lines have provided evidence that more than 95% of multi-exon genes produce alternatively spliced transcripts^11,12^. However, the extent to which alternative transcripts with the potential to encode different proteins are translated has been the subject of considerable debate^63,64^, in large part due to the lack of MS datasets with sufficiently deep coverage. Accordingly, using the high-coverage data generated here, we assessed the proportion of alternatively spliced transcript variants that are detected in the proteome.

To assess the extent to which it is possible to detect splicing within our dataset, we first determined the relative proportions of peptides that fall entirely within exons versus those that span exon–exon junctions. Approximately 30% of identified peptide sequences span junction sequences formed by splicing of protein-coding exons (Supplementary Fig. 7a). Notably, trypsin generates the lowest ratio of junction-spanning versus exon body peptides of all proteases used in this study (~25% versus 28–32%) (Supplementary Fig. 7a). This observation confirms in silico predictions of the limited utility of trypsin alone for detection of spliced junction sequences in shotgun proteomics data^65^. In particular, peptides from trypsin and LysC digestion that fully map within exons have a clear bias which coincides with the first or last amino acids encoded by exons (Supplementary Fig. 8b). Additionally, exon-spanning LysN peptides tend to overlap by a single amino acid at their C termini (Supplementary Fig. 8c). These data are also consistent with a high frequency of lysine residues overlapping splice sites^65^ and illustrate the importance of utilizing additional proteases (chymotrypsin, AspN, GluC and so on) when attempting to detect splice isoforms.

**Fig. 4:**
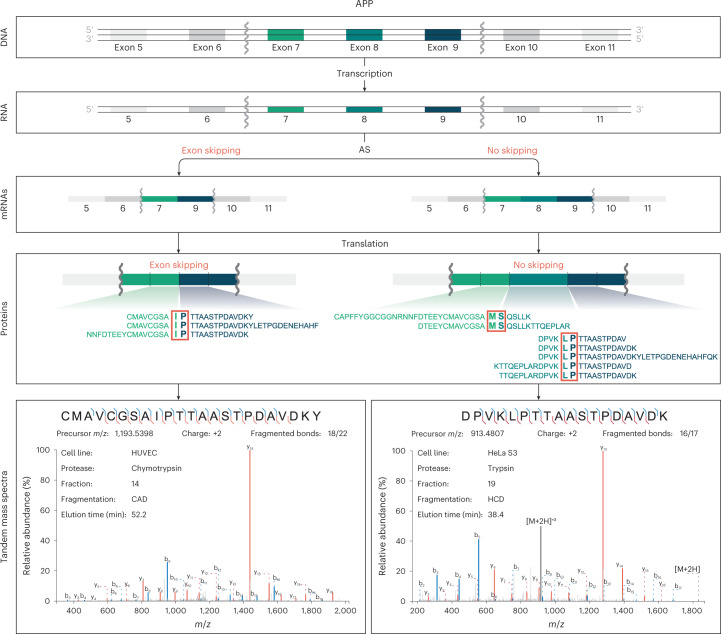
Example of proteomics data corroborating occurrence of an alternative splicing (AS) event in APP. The initial sequential order of exons undergoes transcription. Splicing processing follows, resulting in either 7–9 or 7–8–9 exon combinations. Since all mentioned exons are part of APP’s open reading frame, they have a theoretical possibility to be present and translated into a protein sequence. The multi-enzyme shotgun MS approach described here allows detection of peptides specific to each isoform. Two of 42 total spectra, corroborating these splicing events, are shown.

**Fig. 5:**
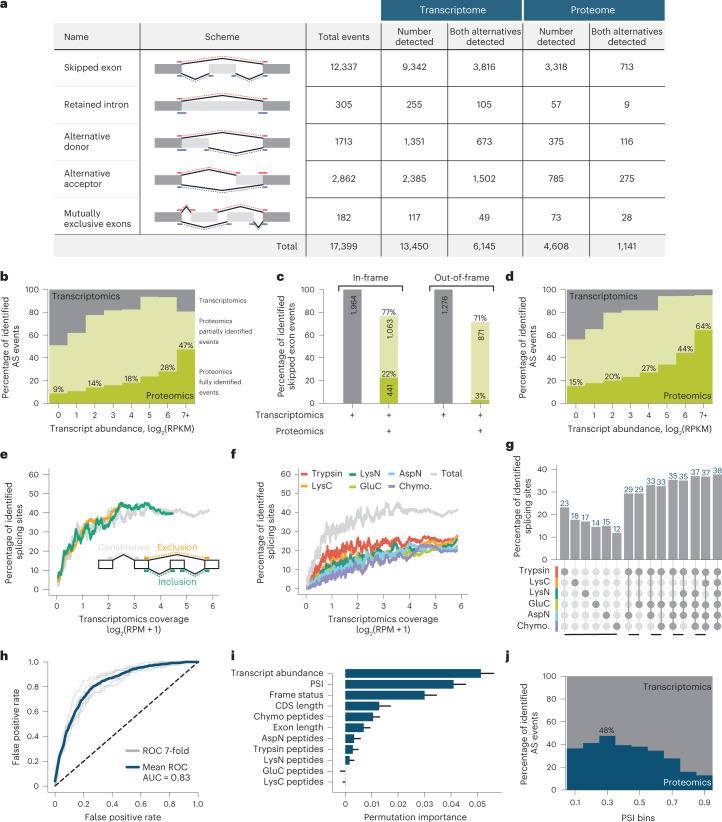
Properties of detected exon skipping AS events. **a**, Summary table of annotated, detected by transcriptomics and proteomics splicing events. AS events are further subdivided into groups with expression evidence for at least one or both alternatives. **b**, Proteomics detection rate of exon skipping AS events as a function of expression. Each gene is grouped by expression level as obtained from RNA-seq data. **c**, Proportions of detected AS events with in-frame or out-of-frame properties. For in-frame AS events, the length of included exon is divisible by 3. It is not the case for out-of-frame AS events which hence result in a frameshift. **d**, The same analysis as in **b** but performed based on frame-preserving isoform events only. **e**, Percentage of MS-identified splicing sites as a function of transcriptional coverage (reads per million, RPM). Three groups of splicing sites are displayed—constitutive (present in all isoforms of a specific gene), exclusion and inclusion splice sites. For more information, see Supplementary Fig. 8. **f**, The same as **e**, but by individual proteases used in this study or all combined (Total). **g**, Splice junction proteomic coverage achieved over all protease combinations. The top two combinations are displayed for 2–5 proteases. Only splice junctions with transcriptomics coverage of more than 1 RPM are included in this analysis. **h**, ROC curve of a binary XGBoost^68^ classifier trained to predict whether AS events are detected or not detected on the proteomics level. **i**, Features ranked by their importance for the XGBoost classifier. The bars and whiskers demonstrate mean and 1 s.d. accordingly. The visualized values were calculated over 100 random shuffles for each parameter. **j**, Proteomics detection rate as a function of percent spliced-in (PSI) value defined by RNA-seq data. AUC, area under the curve.

Figure 4 illustrates our strategy for detection of translated alternative splicing events. In the example provided, alternative splicing of a cassette exon (exon 8) of the Amyloid precursor protein (*APP*) gene is detected by a combination of peptides spanning exons 7 and 9, the junction formed by skipping of the exon, and by peptides spanning exons 7 and 8 or exons 8 and 9, which are formed by inclusion of the exon. In total, we detect 11 unique peptides spanning these three junctions, thus confirming translation of isoforms resulting from inclusion and skipping of the exon. Figure 5a depicts the major classes of alternative splicing events and the detection frequencies of these as they appear in RNA-seq data^49^ generated from all six cell lines analyzed in this study, and the numbers of these events detected at the proteomics level, when considering peptides mapping to one of both possible resulting isoforms (Supplementary Table 3). With a requirement for expression of at least one of two isoforms, we detect 4,608 of 13,450 (34.3%) alternative splicing events (Fig. 5a). Notably, of 6,145 alternative splicing events with RNA-seq expression evidence for both alternatives, we detect 1,141 (18.6%) at the protein level, where junction-spanning peptides representing both alternative isoforms are identified.

Several factors inherently limit the detection of transcript isoforms at the protein level. These include (1) relatively low transcript abundance arising from reduced levels of gene expression; (2) transcript turnover due to nonsense-mediated mRNA decay (NMD), triggered by premature termination codons introduced by frame-shifting alternative splicing events^66^ and other turnover processes; and (3) reduced levels of splicing, as measured using the metric PSI. Exemplifying these limitations, intron retention events, which often result in nuclear retention of transcripts or trigger NMD if the retained intron does not prevent transcript export^67^, are the most rarely detected at the protein level (that is, only 9 of 105). Furthermore, the rate of detection at the proteomics level gradually increases as the corresponding transcript levels for cassette alternative exons increase (Fig. 5b). Moreover, most of the events detected at the proteomics level derive from frame-preserving (that is, in-frame) alternative isoforms (Fig. 5c). Considering only frame-preserving alternative splicing events in relatively abundant transcripts (that is, ≥7 log_2_ RPKM), we observe 64% of alternative spliced events at the protein level (Fig. 5d).

To estimate the possible upper bound detection rates for alternative splicing events at the proteomics level, we compared relative detection rates for alternatively spliced and constitutively spliced junctions in the same RNA transcripts, where constitutively spliced exon–exon junctions are defined as those present in all isoforms of a gene. Importantly, detection rates for constitutive and alternative exon–exon junctions were comparable over a range of transcript levels, in both cases plateauing at approximately 40% of total junctions detected at the highest levels of transcript abundance (Fig. 5e and Supplementary Fig. 9a–f). Consistent with these results, the maximum detection levels require combined data from all six proteases, since each enzyme alone resulted in substantially lower detection levels (Fig. 5f and Supplementary Fig. 9g–i). Additionally, the analysis of all protease combinations shows that nonarginine and nonlysine directed proteases (GluC, AspN and Chymotrypsin) are highly complementary to trypsin in terms of splice site coverage (Fig. 5g).

Finally, to further evaluate factors contributing to the detection of spliced isoforms at the proteomics level, we trained a machine learning binary classifier^68^. Specifically, we classified cassette exon skipping events detected in both proteomics and transcriptomics data versus those events detected solely in the transcriptome. After training on the following properties—transcript abundance, PSI value, exon length, protein coding sequence length, frame-preserving status and a minimum theoretical peptide coverage between isoforms for each studied protease—we evaluated performance using sevenfold cross-validation. This classifier results in 0.83 area under the receiver operating characteristic (ROC) curve (Fig. 5h), which is better than random performance. We next used the permutation importance^69^ to evaluate the importance of each property and to establish the most important ones for influencing proteomic detection of alternative splicing events. The top three most important parameters are transcript abundance, PSI and frame status (Fig. 5i), consistent with the results in Fig. 5b–d.

The PSI ratio reflects the percentage of the total transcript abundance that results in exon inclusion. Since the exon-included isoform contains two junctions for proteomic detection, while the excluded-exon form only contains one, in the case of equally abundant isoforms, exon-inclusion events have double the probability of detection. This situation would result in an optimal PSI for proteomic detection of 33%. This is confirmed in Fig. 5j, where the highest proteomics detection rate for exon exclusion is close to 30%. Note that for extreme PSI values, for example, >0.9, the abundance of the spliced-in isoform is tenfold higher than the splice-out version. This phenomenon likely reduces the overall protein abundance of one isoform, adding to the challenge of its detection.

## Discussion

Here we used six human cell lines, six parallel protease digestions and three MS/MS fragmentation methods to generate over 164 million tandem mass spectra from nearly 2,500 nLC–MS/MS analyses (Fig. 1). Our analysis of the combined data identified over 1 million unique peptides from 17,717 genes encoding protein sequences (Fig. 2). The median protein sequence coverage was 79.2%, representing 8.29 million unique amino acids. The use of proteases that produce sequences complementary to trypsin was particularly important for detecting 2.18 million unique amino acids, increasing the average protein’s sequence coverage by 19%. We conclude that while the use of multiple enzymes only modestly increases protein identification rates, this strategy substantially increases proteomic coverage. A key result from this work is that proteomic coverage gains often come from protein regions with suboptimal trypsin cleavage sites, for example, membrane-spanning domains and splice junctions. Additionally, with the coverage achieved here, we provide evidence that de novo assembly can be accomplished directly from proteomic data, although currently for a limited subset of highly expressed proteins.

We developed informatics tools to allow global detection of nonsynonymous mutations and alternative splicing. Our analysis provides evidence that approximately 73% of nonsynonymous SNPs (that is, SAPs) are translated and present in the proteome. To our knowledge, this is the first proteogenomic study of SAP variants with such depth. This resource now provides a framework to directly study allele-specific expression and address fundamental questions of how mutations impact protein expression and stability^70^. Furthermore, our catalog of expressed SAPs, and the appropriate enzymes and dissociation methods needed to detect them, offers the ability to globally monitor SAPs in both basic and clinical contexts. We note that the ability to detect SAPs in clinical samples could raise privacy concerns^71,72^.

Alternative splicing, which is pervasive at the transcript level, was previously largely undetected at the proteomics level due to the low degree of peptide coverage in most shotgun MS experiments^73^. The failure of proteomics to detect and monitor these events is generally accepted; indeed, it is common practice to report protein isoforms as groups^74^. This shortcoming has limited not only our ability to differentiate protein isoforms but also our knowledge of how splicing impacts the proteome. Here we provide evidence that over half (about 64%) of the frame-preserving splicing events of relatively highly expressed genes detected by transcriptomics are indeed translated and present at the protein level (Fig. 5d), and 22% are detected across the entire expression range (Fig. 5c). Given the highly dynamic nature of protein expression and the challenges of detecting differentially expressed splice variants, we expect these numbers to be underestimates, as evidenced by the lack of full detection of constitutively spliced exon–exon junctions even at the highest levels of peptide coverage (Fig. 5e).

Our study established a compendium of ~25,000 peptides which provide proteomics evidence for ~5,000 splice events. This detection was enabled using multiple proteases (Fig. 5f,g). While trypsin digestion generates peptides of a preferred length and behavior for mass spectrometric detection, it also limits the ability to detect splice junctions. Splice site sequences are inherently biased for lysine codons such that trypsin digestion results in an under-representation of junction-spanning peptides (Supplementary Fig. 7b)^65^. The use of alternative proteases, however, generates a substantial increase in peptides spanning these junctions, approximately doubling their number for MS detection (Fig. 5f). We further confirm additional features of alternative splicing events that limit their detection at the proteomics level. These include intron retention, NMD (which may be triggered by intron retention or other frame-shifting events), and low or high PSI range alternative splicing events (Fig. 5j), which may arise because of splicing regulation or transcript turnover. Our results are largely consistent with the findings of previous ribosome profiling studies, providing evidence that the majority of alternatively spliced junctions overlapping coding sequence in stably expressed transcripts are translated^75^. The results of the present study provide direct evidence that alternative splicing is widespread at the protein level, refuting conclusions of previous studies on MS data with limited coverage generated using trypsin alone^63,64^.

Owing to its scope, depth and coverage, the dataset reported in this study represents a resource to drive future work on the human proteome. To make this peptide catalog accessible, we have created an online resource—deep-sequencing.app. This resource has a gene-centric design, such that one can query any gene and examine the corresponding peptides, SAPs and splicing junctions detected. Beyond providing detailed knowledge of selected genes and their proteoforms, these data could be similarly useful for MS analyses by targeted proteomics and for large-scale machine learning endeavors^76^. For targeted work, our resource provides a global-scale proteomics database of mutations and splice junctions, and the specific peptides that enable their monitoring. For machine learning, it offers over 12 million PSMs from the use of multiple proteases and MS/MS dissociation methods. These data resources will thus enable new insights into unstudied portions of the proteome, potentially offering improved prediction of parameters including peptide detectability, dissociation behavior and chromatographic retention. Finally, these data are expected to facilitate prioritization of SAPs and protein isoforms for future functional studies.

## Methods

### Cell culture and lysis

HeLa S3 cells (CCL-22; ATCC) were grown at 37 °C with 5% CO_2_ in F-12K medium (ATCC) supplemented with 10% FBS and antibiotics. HUVEC cells (CC-2517; Lonza) were grown at 37 °C with 5% CO_2_ in Endothelial Growth Media (EGM) supplemented with EGM Complete Media (Lonza) and antibiotics. HepG2 cells (HB-8065; ATCC) were grown at 37 °C with 5% CO_2_ in EMEM (ATCC) supplemented with 10% FBS and antibiotics. K562 cells (CCL-243; ATCC) were grown at 37 °C with 5% CO_2_ in IMDM (ATCC) supplemented with 10% FBS and antibiotics. GM12878 cells (GM12878 K Order 104598; Coriell Institute for Medical Research) were supplemented with 15% FBS and RPMI-1640 medium (Sigma Aldrich). hESC-1 cells were prepared according to previously published protocols^77^. Cells were collected at >70% confluency through centrifugation at 300*g* for 5 min at 4 °C. The supernatant was removed, and cells were washed with PBS and centrifuged at 300*g* for 5 min at 4 °C. The resulting pellet was stored at −80 °C. Cell pellets were resuspended in lysis buffer containing 8 M urea, 50 mM Tris (pH 8), 5 mM CaCl_2_, 30 mM NaCl, and protease (Roche) and phosphatase (Roche) inhibitor tablets. The pellet was lysed by four rounds of sonication at 4 °C, alternating between 20 s on and 20 s off. Lysate protein concentration was measured by Bicinchoninic acid Protein Assay Kit (Thermo Pierce).

### Digestion

Protein was reduced by addition of 5 mM dithiothreitol and incubation for 45 min at 55 °C. The mixture was cooled to room temperature, followed by alkylation of free thiols by addition of 15 mM iodoacetamide in the dark for 30 min. The alkylation reaction was quenched with 5 mM dithiothreitol. For tryptic digestion, a 1-mg protein aliquot was digested overnight with 20 µg of trypsin (Promega) at room temperature in 1 M urea. For LysC digestion, a 1-mg protein aliquot was digested overnight with 20 µg of LysC (Wako) at room temperature in 4 M urea. For LysN digestion, a 1-mg protein aliquot was digested for 4 h with 20 µg of LysN (Thermo Pierce) at 37 °C in 4 M urea. For GluC digestion, a 1-mg protein aliquot was digested overnight with 25 µg of GluC (Roche) at room temperature in 0.5 M urea. For chymotrypsin digestion, a 1-mg protein aliquot was digested overnight with 12.5 µg of chymotrypsin resuspended in 0.2% formic acid (Promega) in 1 M urea. For digestion with AspN, a 1-mg protein aliquot was incubated with 6 µg of AspN (Roche) at room temperature overnight. Each digest was quenched by the addition of Trifluoroacetic acid and desalted on a 100-mg C_18_ Sep-Pak cartridge (Waters).

### Fractionation

High-pH reversed-phase (RP) fractionation was performed using either a Surveyor LC quaternary pump or a Dionex UltiMate 3000. Fractionation was performed at a flow rate of 1.0 ml min^−1^ using a 5-µm column packed with C18 particles (250 × 4.6 mm^2^, Phenomenex) on a Surveyor LC quaternary pump. Samples were resuspended in buffer A and separated using the following gradient: 0–2 min, 100% buffer A, and separated by increasing buffer B over a 60-min gradient at a flow rate of 0.8 ml min^−1^ (buffer A: 20 mM ammonium formate, pH 10; buffer B: 20 mM ammonium formate, pH 10, in 80% ACN). Flow rate was increased to 1.5 ml min^−1^ during equilibration. Fractionation was performed at a flow rate of 0.45 ml min^−1^ using a 1.7-µm column packed with BEH particles (50 × 1 mm^2^, Waters) on a Dionex Ultimate 3000 pump (Thermo). Samples were resuspended in buffer A and separated by increasing buffer B over a 45-min gradient at a flow rate of 0.45 ml min^−1^ (buffer A: 20 mM ammonium bicarbonate; buffer B: 20 mM ammonium bicarbonate in 80% ACN). Trypsin-digested H1-hESC cells were first fractionated via strong cation exchange fractionation. Peptides were dissolved in 400 μl of strong cation exchange buffer A (5 mM KH_2_PO_4_ and 30% acetonitrile (ACN); pH 2.65) and injected onto a polysulfoethylaspartamide column (9.4 × 200 mm^2^; PolyLC) attached to a Surveyor LC quaternary pump (Thermo Electron) operating at 3 ml min^−1^. Fractions were collected every 2 min starting at 10 min into the following gradient: 0–2 min at 100% buffer A, 2–5 min at 0–15% buffer B (5 mM KH_2_PO_4_, 30% ACN and 350 mM KCl (pH 2.65)) and 5–35 min at 15–100% buffer B. Buffer B was held at 100% for 10 min. Fractions were collected from 8–12 min, 12–14 min, 14–16 min and 16–25 min. Each of these four strong cation-exchange fractions was further fractionated by high-pH RP fractionation on a Surveyor LC quaternary pump, as described above.

### LC–MS/MS

Samples were resuspended in 0.2% formic acid and separated via RP chromatography. Peptides were injected onto an RP column prepared in-house. Approximately 35-cm lengths of 75-μm to 360-μm inner/outer diameter bare-fused silica capillaries, each with a laser pulled electrospray tip, were packed with 1.7-μm diameter, 130-Å pore size, Bridged Ethylene Hybrid C18 particles (Waters). Columns were fitted onto either a nanoAcquity (Waters) or a Dionex (Thermo) and heated to 60 °C using a home-built column heater. Mobile phase buffer A was composed of water and 0.2% formic acid. Mobile phase B was composed of 70% ACN, 0.2% formic acid and 5% dimethylsulfoxide. Each sample was separated over a 100-min gradient, including time for column re-equilibration. Flow rates were set at 300–350 µl min^−1^.

Peptide cations were converted to gas-phase ions by electrospray ionization and analyzed on a Thermo Orbitrap Fusion or a Thermo Orbitrap Lumos (Thermo Fisher Scientific). All fractions were analyzed using HCD. Precursor scans were performed from 300 to 1,500 *m*/*z* at either 60,000 or 120,000 resolution (at 400 *m*/*z*). A 5 × 10^5^ ion count target was used on the Orbitrap Fusion; a 1 × 10^6^ ion count target was used on the Orbitrap Lumos. Precursors selected for MS/MS were isolated at 0.7 Thomson (Th) with the quadrupole, fragmented by HCD with a normalized collision energy of 30 and analyzed using turbo scan in the ion trap. For some analyses, precursors above 500 *m*/*z* were fragmented by HCD using the described conditions, while precursors below 500 *m*/*z* were fragmented by CAD with a normalized collision energy of 30. The maximum injection time for MS/MS analysis was normally set at either 25 or 35 ms, but was set higher for some analyses, with an ion count target of 10^4^. Precursors with a charge state of 2–8 were sampled for MS/MS. Dynamic exclusion time was set at 15 s, with a 10-p.p.m. tolerance around the selected precursor and its isotopes. Monoisotopic precursor selection was turned on. Analyses were performed in top speed mode with either 3- or 5-s cycles.

LysC, LysN, AspN, GluC and chymotrypsin fractions were analyzed using ETD. To maximize identifications, precursor scans were performed from 200 to 800 *m*/*z* at either 60,000 or 120,000 resolution (at 400 *m*/*z*). A 5 × 10^5^ ion count target was used on the Orbitrap Fusion; a 1 × 10^6^ ion count target was used on the Orbitrap Lumos. Precursors selected for MS/MS were isolated at 0.7 Th with the quadrupole. Precursors were fragmented by ETD using custom reaction times; +3: 40 ms, +4: 22 ms, +5: 14 ms, +6: 10 ms, +2: 70 ms. Electron-transfer/higher-energy collision dissociation (EThcD) was performed on +2 precursors, at 25% supplemental activation collision energy. Precursor ions were selected for fragmentation based on charge state in the following order: +3, +4, +5, +6, +2. Fragment ions were analyzed in the ion trap. Dynamic exclusion time was set at 15 s, with a 10-p.p.m. tolerance around the selected precursor and its isotopes. Monoisotopic precursor selection was turned on. Analyses were performed in top speed mode with either 3- or 5-s cycles.

Fractionated peptides from chymotrypsin-catalyzed proteolysis were analyzed using CAD. Precursor scans were performed from 300 to 1,500 *m*/*z* at either 60,000 or 120,000 resolution (at 400 *m*/*z*). A 5 × 10^5^ ion count target was used on the Orbitrap Fusion; a 1 × 10^6^ ion count target was used on the Orbitrap Lumos. Precursors selected for MS/MS were isolated at 0.7 Th with the quadrupole, fragmented by CAD with a normalized collision energy of 30 and analyzed using turbo scan in the ion trap. The maximum injection time for MS/MS analysis was normally set at either 25 or 35 ms, but was set higher for some analyses, with an ion count target of 10^4^. Precursors with a charge state of 2–8 were sampled for MS/MS. Dynamic exclusion time was set at 15 s, with a 10-p.p.m. tolerance around the selected precursor and its isotopes. Monoisotopic precursor selection was turned on. Analyses were performed in top speed mode with either 3- or 5-s cycles.

### Protein identification

The 2,491 raw files were simultaneously analyzed by database search to identify proteins and peptides using the Andromeda search engine^50^ inside MaxQuant (v.1.5.7.5)^51,52^. Searches were performed against the following protein sequence databases: UniProt canonical (release 2017_02; UP000005640_9606), UniProt isoform (UP000005640_9606_additional), Ensembl canonical (release 86; GRCh38.pep.all), Ensembl isoform (GRCh38.pep.abinitio). Searches used the default precursor mass tolerances (20 p.p.m. first search and 4.5 p.p.m. main search) and a product mass tolerance of 0.35 Da. The in silico digest was set to specific cleavage and a maximum of two missed cleavages for all proteases, except chymotrypsin, where up to four missed cleavages were allowed. Parameters for each protease (LysC, LysN, chymotrypsin, AspN, GluC and trypsin) were set in groups. The fixed modifications specified were carbamidomethylation of cysteine residues and variable modifications were oxidation of methionine and acetylation of protein N terminus. PSMs and protein groups were both sequentially filtered to a 1% FDR over the whole dataset, resulting in detection of 12,151,708 forward PSMs (7,469 reverse PSMs; 0.06% FDR), 1,119,510 forward peptides (4,486 reverse peptides; 0.4% FDR) and 17,717 proteins (176 reverse proteins; 0.99% FDR). Note, PSMs that match only to protein groups that do not pass the protein-level FDR filtering are not present in the output tables, resulting in lower than the initially specified 1% PSM FDR (0.06%). Protein groups were filtered for ‘Only identified by site’, ‘Reverse’ and ‘Contaminant’. Gene locus information was mapped to majority protein identifications with Human Gene Nomenclature Database identifications from UniProt and Ensembl BioMart.

### Protein coverage calculation

Sequence coverage for various subsets of runs was calculated with a custom C# application. For each row in the MaxQuant proteinGroups.txt output, all associated peptides were retrieved from peptides.txt. For each peptide, it was first determined whether it was found in this subset of runs, using the experiment-based PSM count columns in peptides.txt. If so, the sequence was searched for all occurrences in the sequence of the first major protein of the protein group, ignoring enzyme specificity. A list of unique amino acid residues observed was maintained across all peptides, and at the end the number of residues in the list was divided by the total number of residues in the major protein sequence. Whenever possible, sequence coverages obtained in this manner were compared with those computed by MaxQuant and included in proteinGroups.txt, and the agreement was excellent. The console C# code is located at https://github.com/cwenger/cwenger.github.io/tree/master/MaxQuantAnalyzer.

### Spectra visualization and annotation

All presented spectra were annotated and visualized with a web-based Interactive Peptide Spectra Annotator^78^. Two spectra shown in Fig. 4 have the following Universal Spectrum Identifiers—mzspec:PXD024364:20160115_alr_CompleteHumanProteome_HUVEC_chymo_CAD_fr14:scan:50088:CMAVCGSAIPTTAASTPDAVDKY/2 (left side) and mzspec:PXD024364:HeLaS3_trypsin_19_140824180249:scan:34854:DPVKLPTTAASTPDAVDK/2 (right side).

### De novo proteome assembly

The PSMs were extracted from the evidence.txt file and filtered by ‘Potential contaminant’ and ‘Reverse’. Each PSM was reverse translated into nucleotide sequence with a nondegenerate codon table and written into a FASTA file as input to SOAPdenovo. The SOAPdenovo config file parameters were set to default except for maximal read length to 150. SOAPdenovo-Trans-31mer was run with *k*-mer length 23 (at least 8 amino acids) and minimum contig length 100 (at least 34 amino acids). Scaffolds from the assembly were matched back to the proteome sequences using brute force string matching.

### RNA-seq data and analysis

The paired RNA-seq data for HeLa S3/HUVEC/HepG2/K562/GM12878/hESC are a part of the ENCODE dataset^49^ and were downloaded from SRA (SRP014320). Raw reads were filtered using trimmomatic (v.0.36) using default parameters for paired-end data. Filtered reads were mapped to the human reference genome GRCh38 (Ensemble release 91) using STAR aligner (v.2.5.3a). Further processing—sorting, converting from SAM to BAM format and indexing—was done using SAMtools (v.1.6).

To compare proteomics and transcriptomics data (Fig. 3b), raw reads per gene were counted in Perseus (v.1.6.14.0)^79^, and rows were logarithmized with pseudocount 1 and normalized by *z*-scoring for each experiment independently. Intensity-based absolute quantification values from the standard proteomics search were summed for each cell line (through fractions, fragmentation methods and proteases), logarithmized, *z*-scored for each cell line independently and imputed by replacing missing values from the normal distribution (width = 0.3, down shift = 1.8), separated for each cell line. After joining the two tables, genes with both proteomics and transcriptomics data were used for the principal component analysis plot. Component 1 (accounting for 27.8% of the variance) was not used because it explains the difference between proteomics and transcriptomics data.

### Mutation analysis—transcriptomics

Nonsynonymous mutations were extracted from RNA-seq data of all studied cell lines using the ‘Variation extraction’ tool in MaxQuant (Tools/Variation extraction; Supplementary Fig. 4)^59^. This tool reports in a fasta file all nonsynonymous mutations that pass a list of filters: total reads depth should more than or equal to 10; number of reads with mutations should be more than or equal to 5; the frequency of reads with mutations to overall depth should be more than or equal to 15%; the base quality, as well as the mapping quality, should be more than or equal to 13, which automatically filters out multi-mapped reads. The ‘Variation extraction’ tool generates, amongst many output files, a *protein.fa* file with all annotated ‘protein_coding’ sequences as well as information about nonsynonymous mutations in a header for each sequence.

### Mutation analysis—proteomics

To enable MaxQuant to use the specified mutations, one has to add the fasta file into the ‘Fasta files’ tab (Global Parameters/Sequences/Fasta files) and change the ‘Variation mode’ parameter to ‘Read from fasta file’^59^. In the MaxQuant output ‘peptides.txt’ file an additional column such as ‘Mutated’ and ‘Mutation names’ columns will be created. The ‘Mutated’ column reports ‘No’ if one peptide comes from the reference proteome (without mutations), ‘Yes’ if a peptide results from mutation inclusion and ‘Mixed’ if one can find peptides in the reference as well as mutated proteomes. The ‘Mutation names’ stands for a list of involved mutations.

### Splicing analysis—transcriptomics and proteomics

The analysis of alternative splicing is based on the gene graph structure, where nodes represent the beginnings and the ends of exons, and edges correspond to exon–exon junctions as well as connections within an exon. Each splicing event in this graph is a local subgraph with multiple paths; however, all paths start from the same node and finish on the same downstream node. It is important to point out that one path can consist of several isoforms. The algorithm is adapted from ref. ^80^. To use the same approach for proteomics, protein coordinates of peptides were converted to genome locations, taking into account the intron–exon structure of genes. The modified version of the algorithm is available as a plugin for Perseus software (Supplementary Fig. 6).

### Binary classification of alternative splicing events

The binary classification was conducted with XGBoost python package (v.1.5.0)^68^. The optimum set of learning parameters has been estimated using a grid search (RandomizedSearchCV function from sklearn package) with the sevenfold cross-validation technique and area under the ROC curve as a performance metric. The selected parameters are listed as follow—learning rate: 0.05; L1 regularization weight: 1.15; L2 regularization weight: 4.0; minimum child weight: 2.0; maximum depth: 3; minimum loss reduction (gamma): 2.0; subsample ratio of columns: 0.3; subsample ratio of the training instances: 0.65; scale positive weight: 4.44.

### Reporting summary

Further information on research design is available in the Nature Portfolio Reporting Summary linked to this article.

## Online content

Any methods, additional references, Nature Portfolio reporting summaries, source data, extended data, supplementary information, acknowledgements, peer review information; details of author contributions and competing interests; and statements of data and code availability are available at 10.1038/s41587-023-01714-x.

### Supplementary information

Supplementary Information Supplementary Figs. 1–9.

Reporting Summary

Supplementary Table Supplementary Table 1. Summary of cross-mapping of neXtProt and detected protein accessions. Supplementary Table 2. Summary of all mutations detected in the proteomics and transcriptomics data. Supplementary Table 3. Summary of all splicing events detected in the proteomics and transcriptomics data.

## Data Availability

All raw mass spectrometry data files and MaxQuant output from the standard search have been deposited to the ProteomeXchange Consortium (http://proteomecentral.proteomexchange.org) via the MassIVE^8^ partner repository with the dataset identifier PXD024364. Profiled protein and transcript variants are compiled in the following location: https://deep-sequencing.app.

## Code availability

All code used for visualization is available via the link https://github.com/coongroup/DeepProteomeSequencing-Software.
