# Supplementary material for: Global detection of human variants and isoforms by deep proteome sequencing
Source: Nat Biotechnol. 2023 Mar 23;41(12):1776–86. doi: 10.1038/s41587-023-01714-x (PMC10713452; doi:10.1038/s41587-023-01714-x)
Supplement: Supplementary file 1 — Supplementary Figs. 1–9. [file 41587_2023_1714_MOESM1_ESM.pdf]

nature biotechnology

Article

https://doi.org/10.1038/s41587-023-01714-x

Global detection of human variants and isoforms by deep proteome sequencing

In the format provided by the authors and unedited

Figure S1

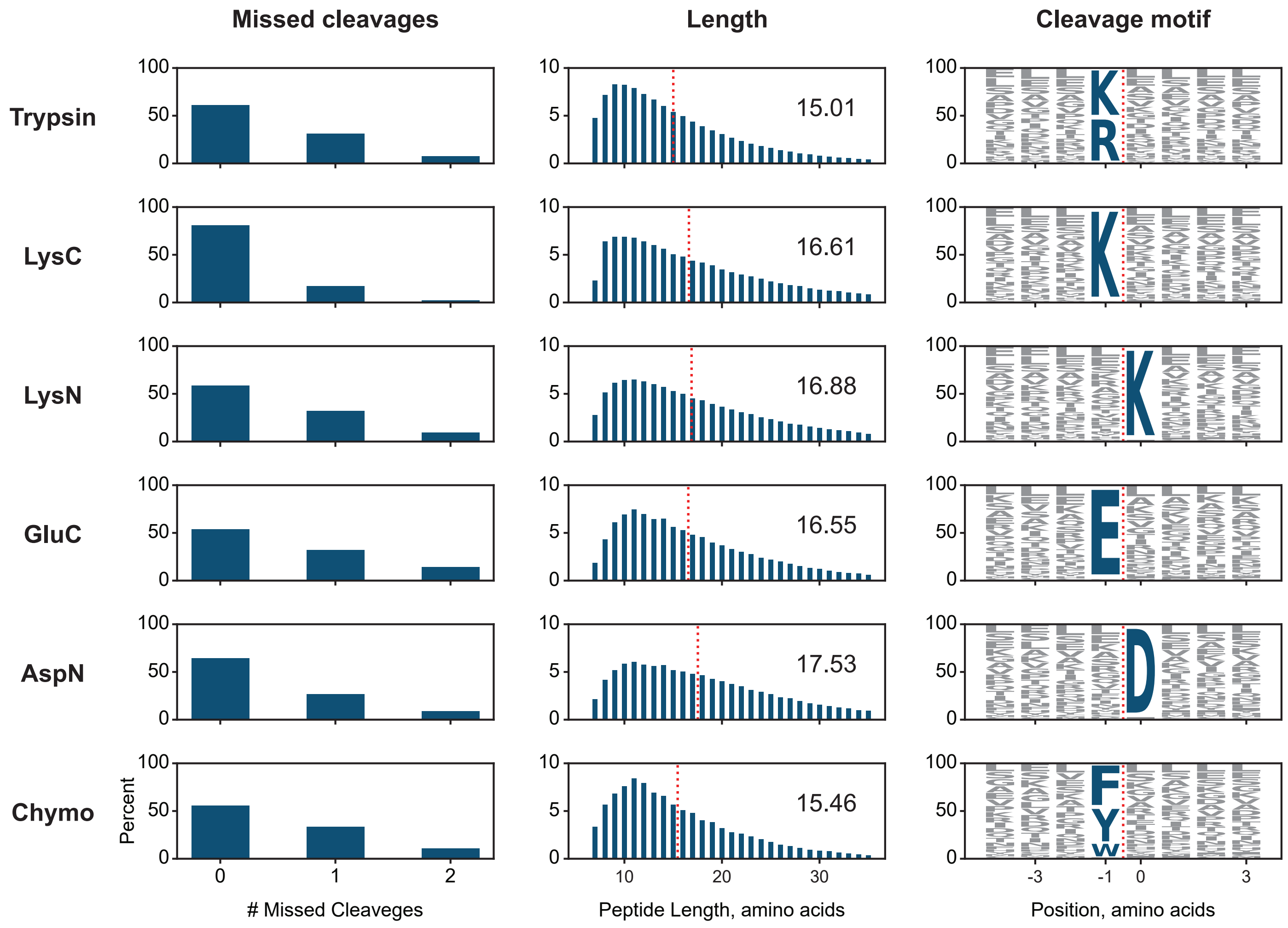

Figure S1. Properties of detected peptides. The left column of histograms shows a relative distribution of missed cleavages over six protease digests, the middle column - relative distribution of detected peptides (red line indicates an average value, which is also stated as a number), the right column - an occurrence of amino acids around the cleavage site (labeled as a red line).

Figure S2

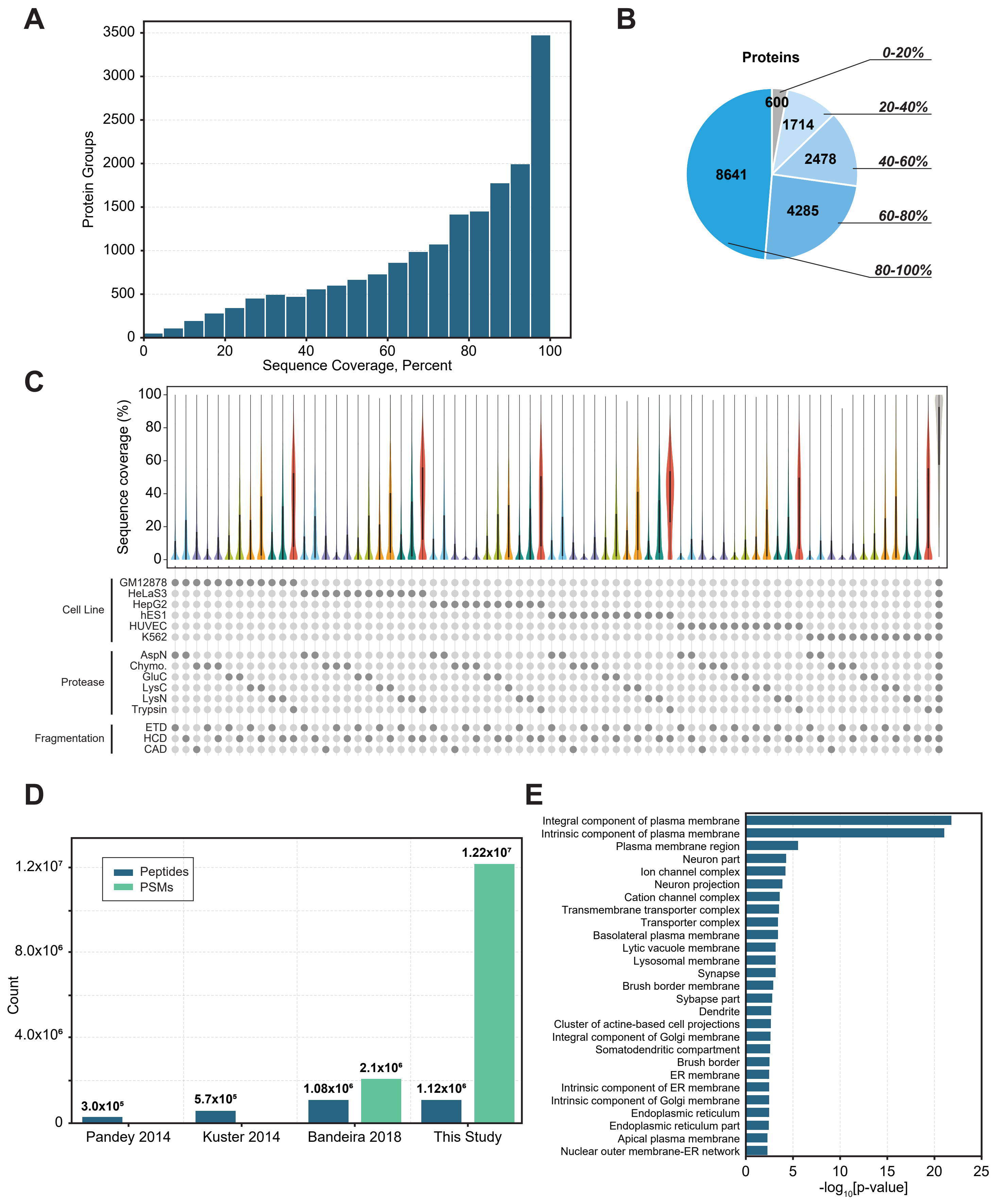

Figure S2. Protein sequence coverage of all identified proteins. A, Histogram showing the number of protein groups binned by observed sequence coverage. **B**, Pie chart showing the number of proteins observed in each of five 20% bins of sequence coverage. **C**, Series of violin plots for all measured combinations of cell lines, proteases, and fragmentation methods. **D**, A number of reported peptides and peptide spectral matches (PSMs) across large-scale proteomics studies. **E**, Cellular component gene ontology analysis of proteins with sequence coverage less than 25% are significantly enriched for membrane proteins accordingly to the Fisher's exact test.

Figure S3

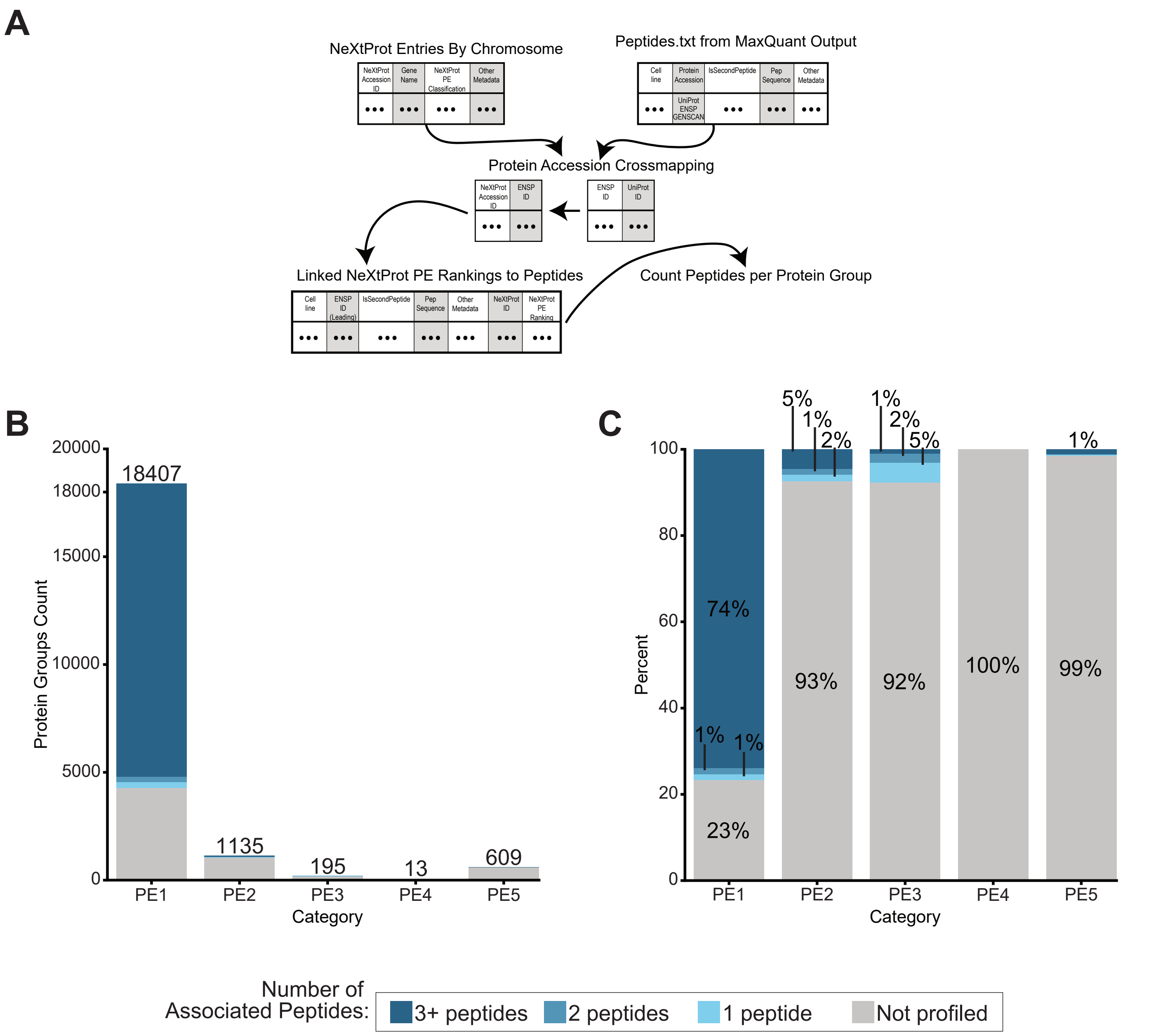

Figure S3. Comparison with the neXtProt annotation. A, The current release of neXtProt (October 2022) was downloaded and cross-mapped to peptides profiled in this study by first converting any proteins demarked by UniProt identifiers to Ensembl Protein identifiers. UniProt to ENSP mapping was obtained from BioMart. Next, Ensembl protein identifiers were mapped to neXtProt accession values via the mapping scheme provided in the October 2022 release. Finally, the number of peptides per neXtProt group were summed across all cell lines used in this study. **B**, Unique neXtProt proteins delineated by protein existence (PE) rank colored by the number of mapped peptides detected in this study. **C**, The relative proportion within each PE rank of neXtProt proteins with 0, 1, 2, or 3+ mapped peptides.

Figure S4

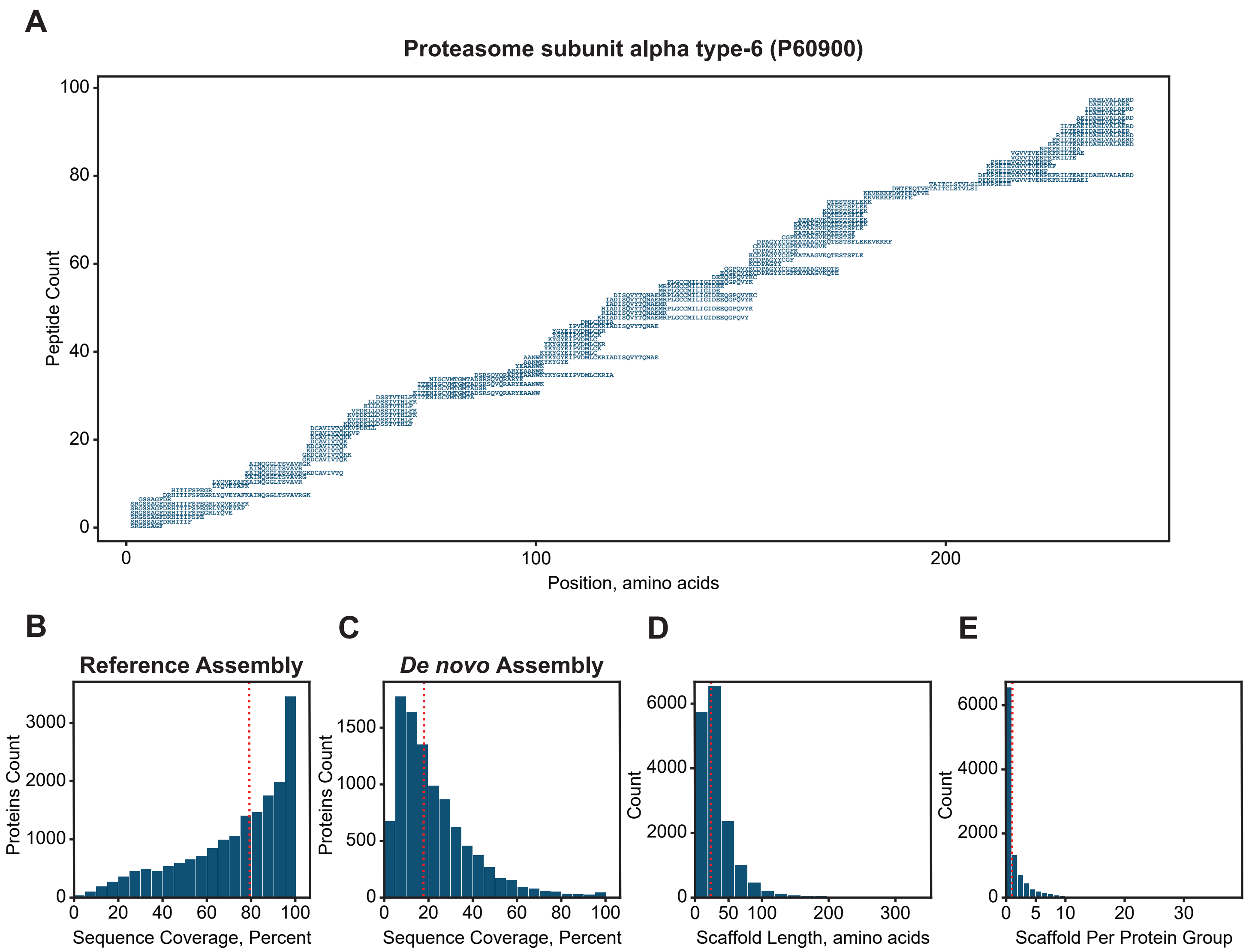

Figure S4. *De novo* assembly of proteins. A, Plot of observed 98 peptide sequences from Proteasome subunit alpha type-6 (UniProt ID: P60900) that were *de novo* assembled to achieve 100% protein coverage. **B**, Histogram of proteins identified by the standard MaxQuant database search binned by sequence coverage with a vertical line at the median coverage. **C**, Histogram of proteins identified by the de novo assembly with a vertical line showing the median coverage. **D**, Length distribution of scaffolds from the de novo assembly. **E**, Number of scaffolds matched to each protein in the de novo assembly.

Figure S5

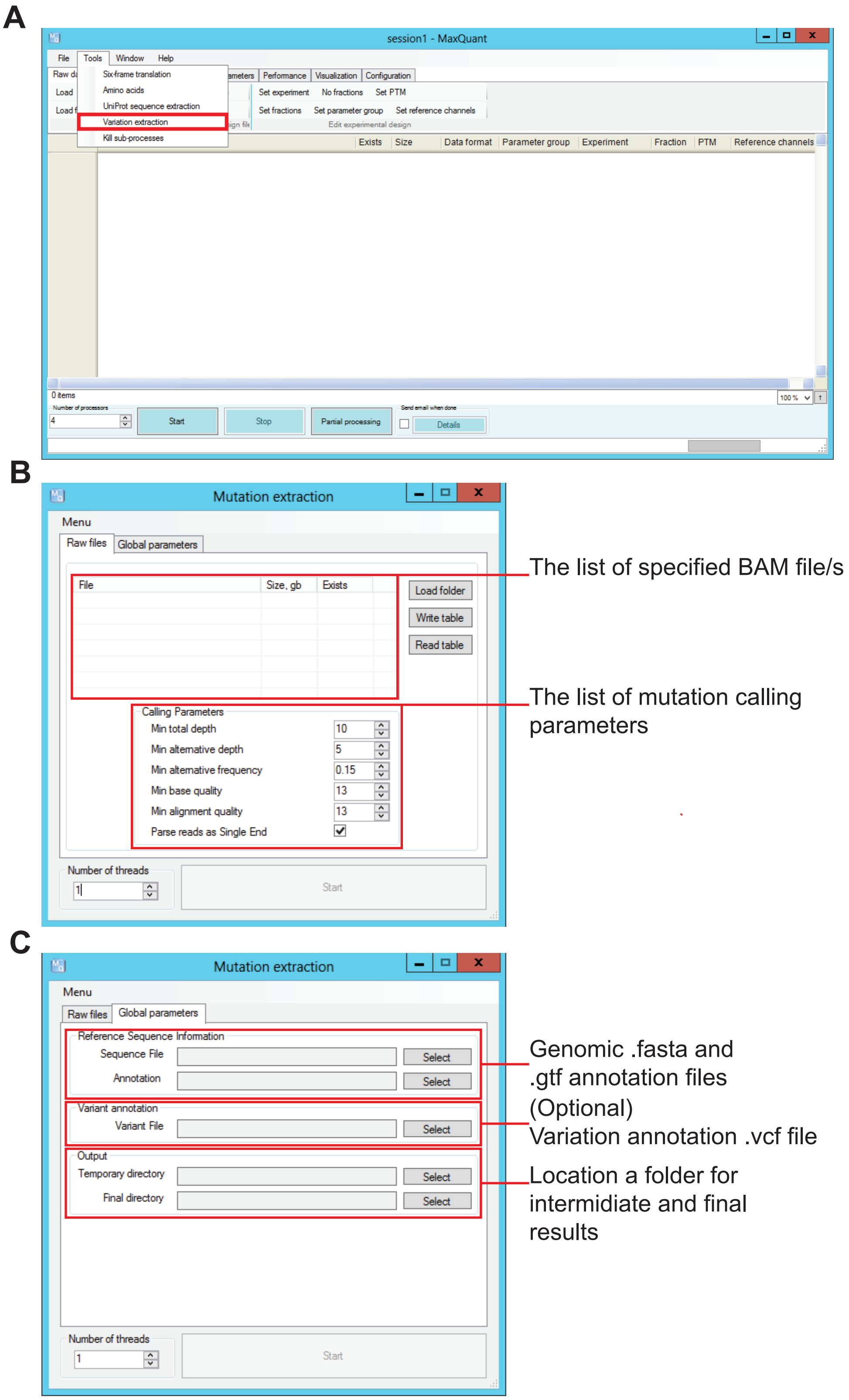

Figure S5. How to perform a variant extraction in MaxQuant. A, Open MaxQuant and follow the “Tools/- Variant extraction” tab. **B**, Specify a list of BAM files with NGS data (RNA-seq, WGS, WES), and if needed, change mutation calling parameters. **C**, Specify location of genomic DNA sequence and genome annotation. Additionally, define folders for temporary and final files.

Figure S6

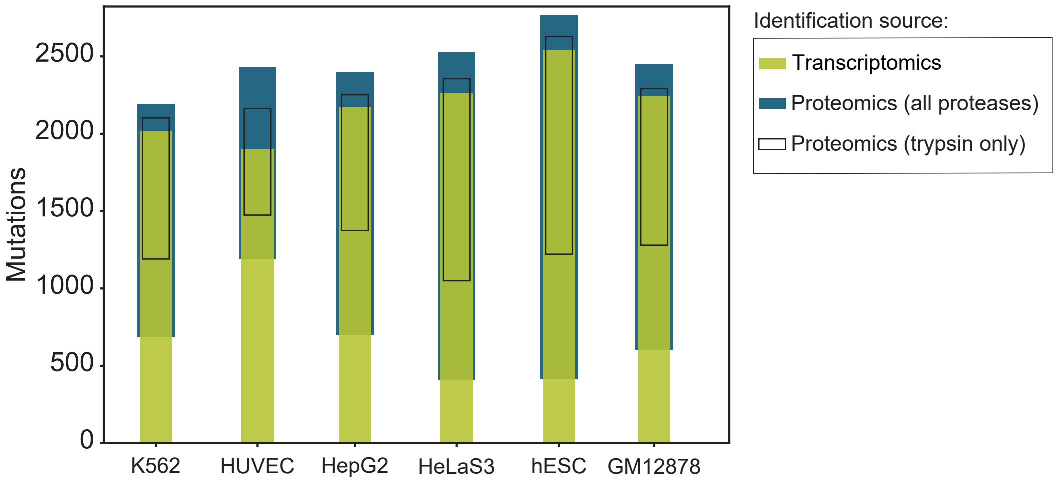

Figure S6. Properties of detected single amino acids polymorphisms. A number of detected SAPs across cell lines and two omics – transcriptomics and proteomics. The SAPs detected with proteomics are further subdivided into ones observed in digests with trypsin or all proteases combined.

Figure S7

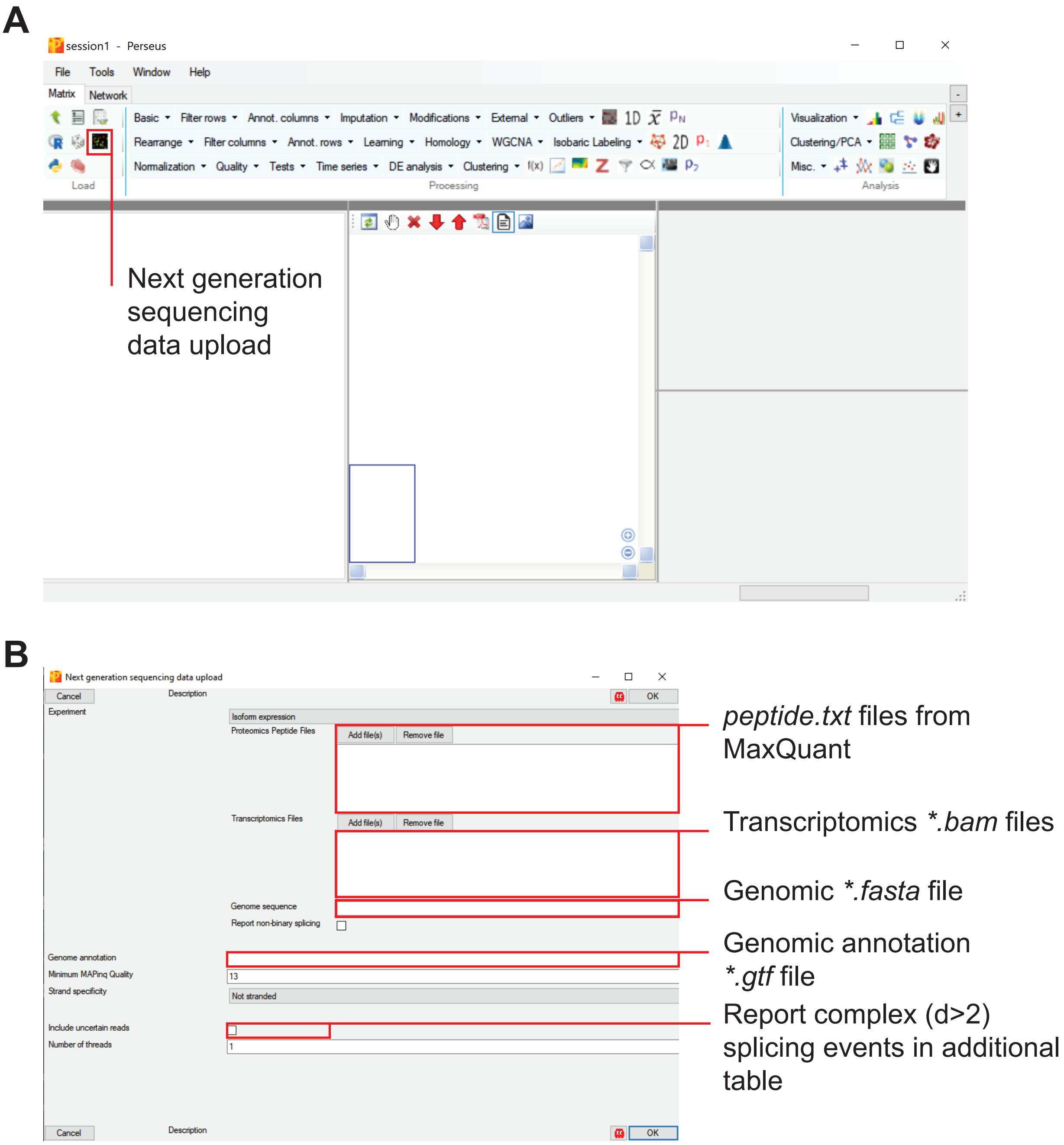

Figure S7. Hot to detect alternative splice events jointly for proteomics and transcriptomics data in Perseus. A, Open Perseus software and follow to “Load/NGS data upload” activity. **B**, Specify the location of peptide.txt files from MaxQuant, transcriptomics BAM files, genome DNA sequence, and annotation.

Figure S8

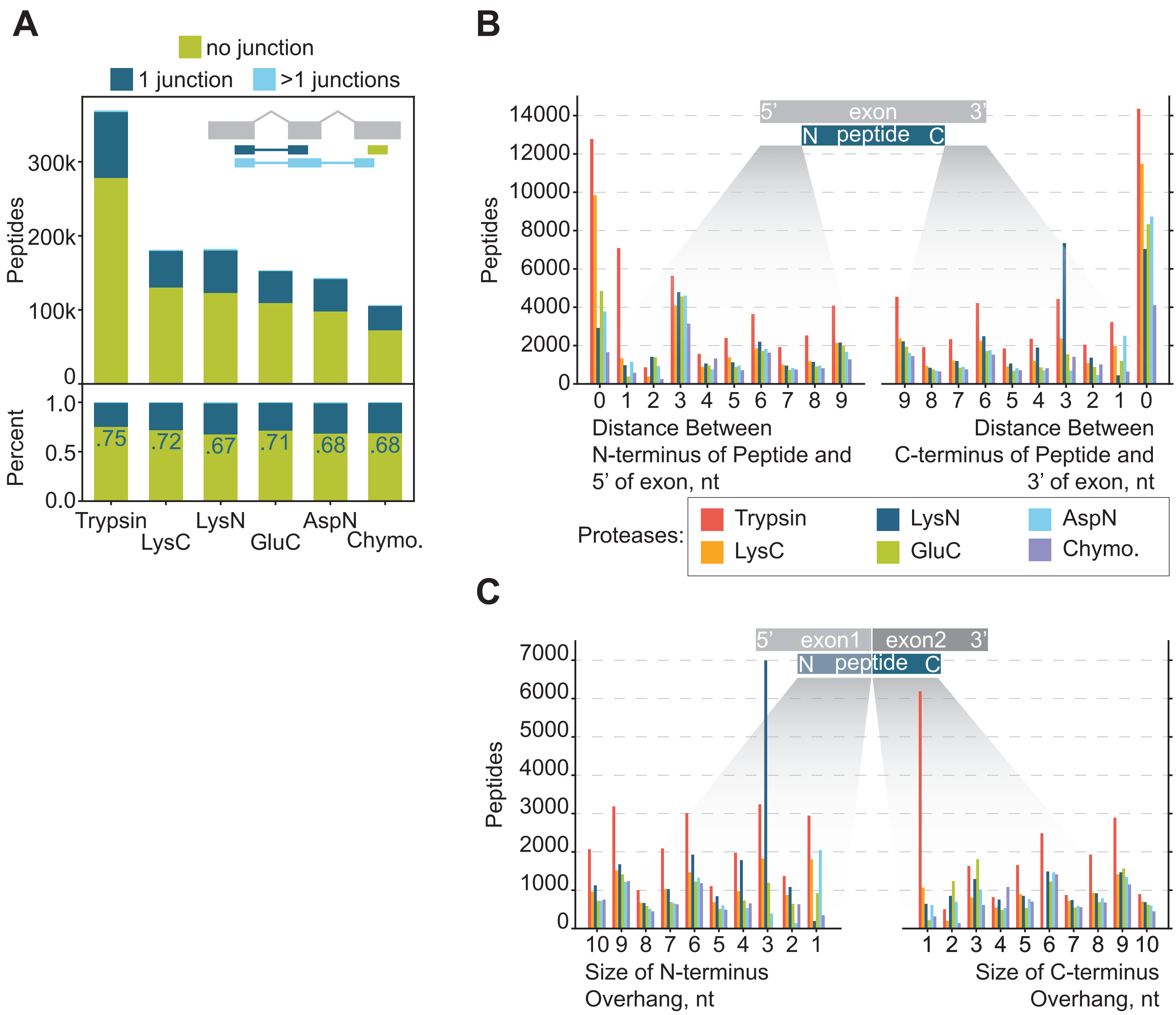

Figure S8. Properties of MS detected peptides proximal to exon boundaries. A, The absolute and relative amounts of detected peptides across multiple proteases. Each set of peptides consists of ones that are mappable to one exon exclusively (no junction), to two exons (one junction), and the rest (more than one junction). **B**, Distribution of peptides over a distance to 5' (left) and 3' (right) end of an exon in nucleotides. **C**, Distribution of peptides with at least one junction over a distance to a splicing site - N-terminus (left) and C-terminus (right) overhang. Both **B** and **C** show distributions across multiple proteases encoded by color.

Figure S9

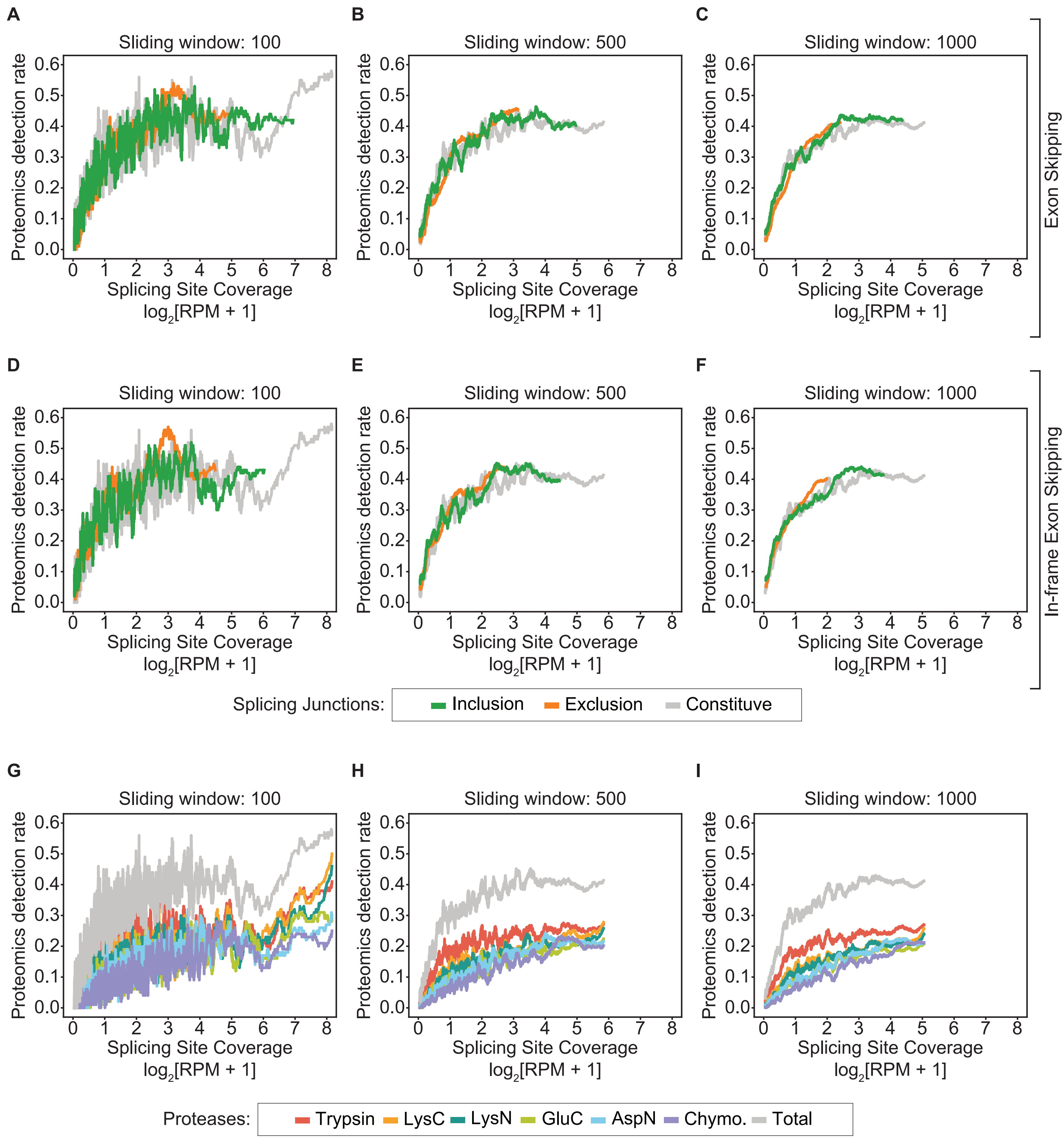

Figure S9. Properties of MS detected peptides spanning spliced exon-exon junctions. A-F, Percent of MS identified splice junctions as a function of transcriptional coverage, measured as logarithm of read count (reads per million - RPM). Splice junctions are further subdivided into constitutive sites, i.e., present in all isoforms of specific genes, and exclusion/inclusion sites, involved in exon skipping alternative splicing. Figures **A-C** demonstrate statistics for all exon skipping events, but **D-F** – for in-frame exon skipping events. The percentage of identified splicing sites was calculated among events sorted by transcription coverage using sliding windows of various lengths - 100 (**A** and **D**), 500 (**B** and **E**), and 1000 (**C** and **F**) events. Note that figure **E** is identical to **Figure 5D**. **G-I,** the same as **D-F**, but for each protease used in this study, or all combined (Total). Note that figure **H** is identical to **Figure 5E**.
