# Supplementary material for: Global detection of human variants and isoforms by deep proteome sequencing
Source: Nat Biotechnol. 2023 Mar 23;41(12):1776–86. doi: 10.1038/s41587-023-01714-x (PMC10713452; doi:10.1038/s41587-023-01714-x)
Supplement: Supplementary file 2 — Reporting Summary [file 41587_2023_1714_MOESM2_ESM.pdf]

nature portfolio

Corresponding author(s): Jürgen Cox and Joshua J. Coon

Last updated by author(s): Feb 10, 2023

Reporting Summary

Nature Portfolio wishes to improve the reproducibility of the work that we publish. This form provides structure for consistency and transparency in reporting. For further information on Nature Portfolio policies, see our Editorial Policies and the Editorial Policy Checklist.

Statistics

For all statistical analyses, confirm that the following items are present in the figure legend, table legend, main text, or Methods section.

n/a	Confirmed	
☐	☒	The exact sample size (*n*) for each experimental group/condition, given as a discrete number and unit of measurement
☐	☒	A statement on whether measurements were taken from distinct samples or whether the same sample was measured repeatedly
☐	☒	The statistical test(s) used AND whether they are one- or two-sided<br>*Only common tests should be described solely by name; describe more complex techniques in the Methods section.*
☒	☐	A description of all covariates tested
☐	☒	A description of any assumptions or corrections, such as tests of normality and adjustment for multiple comparisons
☐	☒	A full description of the statistical parameters including central tendency (e.g. means) or other basic estimates (e.g. regression coefficient) AND variation (e.g. standard deviation) or associated estimates of uncertainty (e.g. confidence intervals)
☐	☒	For null hypothesis testing, the test statistic (e.g. *F*, *t*, *r*) with confidence intervals, effect sizes, degrees of freedom and *P* value noted<br>*Give P values as exact values whenever suitable.*
☒	☐	For Bayesian analysis, information on the choice of priors and Markov chain Monte Carlo settings
☒	☐	For hierarchical and complex designs, identification of the appropriate level for tests and full reporting of outcomes
☐	☒	Estimates of effect sizes (e.g. Cohen's *d*, Pearson's *r*), indicating how they were calculated

Our web collection on statistics for biologists contains articles on many of the points above.

Software and code

Policy information about availability of computer code

Data collection	MaxQuant 1.5.7.5
Data analysis	Perseus 1.6.14.0<br>Trimmomatic 0.36<br>STAR aligner 2.5.3a<br>SAMtools 1.6<br>MaxQuantAnalyzer https://github.com/cwenger/cwenger.github.io/tree/master/MaxQuantAnalyzer<br>XGBoost 1.5.0<br>Python 3.6<br>SIFT 6.2.1<br>PolyPhen-2 2.2.3<br><br>All code used for visualization is available via the link https://github.com/coongroup/DeepProteomeSequencing-Software.

For manuscripts utilizing custom algorithms or software that are central to the research but not yet described in published literature, software must be made available to editors and reviewers. We strongly encourage code deposition in a community repository (e.g. GitHub). See the Nature Portfolio guidelines for submitting code & software for further information.

Data

Policy information about availability of data

All manuscripts must include a data availability statement. This statement should provide the following information, where applicable:

- Accession codes, unique identifiers, or web links for publicly available datasets
- A description of any restrictions on data availability
- For clinical datasets or third party data, please ensure that the statement adheres to our policy

All raw mass spectrometry data files and MaxQuant output from the standard search have been deposited to the ProteomeXchange Consortium (http://proteomecentral.proteomexchange.org) via the MassIVE partner repository with the dataset identifier PXD024364. Human protein fasta files have been retrieved from Uniprot (release 2017_02; canonical UP000005640_9606.fa and additional file UP000005640_9606_additional.fa) and from Ensemble (release 86; Homo_sapiens.GRCh38.pep.all.fa and Homo_sapiens.GRCh38.pep.abinitio.fa). The paired RNA-seq data for HeLaS3/HUVEC/HepG2/K562/GM12878/hESC ares a part of the ENCODE dataset and was downloaded from SRA (SRP014320).

Human research participants

Policy information about studies involving human research participants and Sex and Gender in Research.

Reporting on sex and gender	n/a
Population characteristics	n/a
Recruitment	n/a
Ethics oversight	n/a

Note that full information on the approval of the study protocol must also be provided in the manuscript.

Field-specific reporting

Please select the one below that is the best fit for your research. If you are not sure, read the appropriate sections before making your selection.

☒ Life sciences ☐ Behavioural & social sciences ☐ Ecological, evolutionary & environmental sciences

For a reference copy of the document with all sections, see nature.com/documents/nr-reporting-summary-flat.pdf

Life sciences study design

All studies must disclose on these points even when the disclosure is negative.

Sample size	No prior sample size calculation was performed. 6 cell lines have been chosen for this study since the initial version of ENCODE focused on the same list of cell lines.
Data exclusions	No data were excluded from the analysis.
Replication	No replication LC-MS measurements have been conducted since identification maximization was a priority in this study.
Randomization	All cell lines have been LC-MS measured sequentially. No randomization has been applied in this study.
Blinding	Sample preparation and LC-MS measurements have been performed by one investigator (Alicia L. Richards). No blinding has been applied.

Reporting for specific materials, systems and methods

We require information from authors about some types of materials, experimental systems and methods used in many studies. Here, indicate whether each material, system or method listed is relevant to your study. If you are not sure if a list item applies to your research, read the appropriate section before selecting a response.

Materials & experimental systems

n/a	Involved in the study
☒	☐ Antibodies
☐	☒ Eukaryotic cell lines
☒	☐ Palaeontology and archaeology
☒	☐ Animals and other organisms
☒	☐ Clinical data
☒	☐ Dual use research of concern

Methods

n/a	Involved in the study
☒	☐ ChIP-seq
☒	☐ Flow cytometry
☒	☐ MRI-based neuroimaging

Eukaryotic cell lines

Policy information about cell lines and Sex and Gender in Research

Cell line source(s)	HeLa S3 cells (ATCC CCL-22; ATCC, Manassas, VA); HUVEC cells (Lonza CC-2517; Lonza, Walkersville, MD); HepG2 cells (ATCC HB-8065; ATCC); K562 cells (ATCC CCL-243; ATCC); GM12878 cells (GM12878 K Order 104598; Coriell Institute for Medical Research, Camden, NJ); hESC-1 cells were prepared by James Thomson laboratory according to previously published protocols (PMID: 21983960).
Authentication	The cell lines used were not authenticated.
Mycoplasma contamination	The cell lines were not tested for mycoplasma contamination.
Commonly misidentified lines (See ICLAC register)	No commonly misidentified cell lines were used in the study.
